# Supplementary material for: Plasmidome of Salmonella enterica serovar Infantis recovered from surface waters in a major agricultural region for leafy greens in California
Source: PLoS One. 2024 Dec 30;19(12):e0316466. doi: 10.1371/journal.pone.0316466 (PMC11684603; doi:10.1371/journal.pone.0316466)
Supplement: S1 Table — (DOCX) [file pone.0316466.s001.docx]

**S1 Table. *Salmonella enterica* serovar Infantis strains screened for plasmids in this study.**

| **Strain** | **Serotype** | **Sample Date^a^** | **Plasmid band size range** |
| --- | --- | --- | --- |
| RM15962 | Infantis | 01/23/2012 | ND^b^ |
| RM16752 | Infantis | 7/19/2012 | 74-100 kb |
| RM 17883 | Infantis | 12/3/2012 | 74-100 kb |
| RM 17884 | Infantis | 12/3/2012 | 74-100 kb |
| RM 17885 | Infantis | 12/3/2012 | 74-100 kb |
| RM 20997 | Infantis | 12/3/2012 | ND |
| RM 21253 | Infantis | 3/17/2012 | ND |
| RM 17050 | Infantis | 3/20/2013 | 74-100 kb |
| RM 17053 | Infantis | 3/20/2013 | ND |
| RM 17691 | Infantis | 5/29/2013 | ND |
| RM 17931 | Infantis | 1/2/2013 | ND |
| RM 18147 | Infantis | 5/29/2013 | 74-100 kb |
| RM 18148 | Infantis | 5/29/2013 | 74-100 kb |
| RM 18154 | Infantis | 6/12/2013 | ND |
| RM 18281 | Infantis | 11/20/2013 | >100 kb |
| RM 18287 | Infantis | 11/20/2013 | ND |
| RM 18424 | Infantis | 10/23/2013 | ND |
| RM 21049 | Infantis | 7/8/2013 | ND |
| RM 18410 | Infantis | 3/1/2014 | 74-100 kb |
| RM 18512 | Infantis | 4/2/2014 | 74-100 kb |
| RM 18513 | Infantis | 4/15/2014 | 74-100 kb |
| RM 18520 | Infantis | 4/15/2014 | ND |
| RM 18673 | Infantis | 4/30/2014 | ND |
| RM 18841 | Infantis | 7/22/2014 | ND |
| RM 21071 | Infantis | 4/2/2014 | 74-100 kb |
| RM 21074 | Infantis | 8/7/2014 | ND |
| RM 21082 | Infantis | 9/3/2014 | 74-100 kb |
| RM 21085 | Infantis | 9/3/2014 | 74-100 kb |
| RM 21087 | Infantis | 9/3/2014 | 74-100 kb |
| RM 21154 | Infantis | 10/15/2014 | 74-100 kb |
| RM 19091 | Infantis | 3/22/2016 | ND |
| RM 19096 | Infantis | 3/22/2016 | ND |
| RM 19102 | Infantis | 3/22/2016 | ND |
| RM 21474 | Infantis | 7/21/2016 | ND |

^a^Collection date of the water/sediment sample from which the strain was isolated.

^b^ND, Plasmid not detected in gel.
