# Supplementary material for: Plasmidome of Salmonella enterica serovar Infantis recovered from surface waters in a major agricultural region for leafy greens in California
Source: PLoS One. 2024 Dec 30;19(12):e0316466. doi: 10.1371/journal.pone.0316466 (PMC11684603; doi:10.1371/journal.pone.0316466)
Supplement: Attachment — Submitted filename: PONE-D-24-42911.pdf [file pone.0316466.s002.pdf]

PLOS ONE

Plasmidome of Salmonella enterica serovar Infantis recovered from surface waters in a major agricultural region for leafy greens in California

--Manuscript Draft--

**Manuscript Number:**	PONE-D-24-42911
**Article Type:**	Research Article
**Full Title:**	Plasmidome of Salmonella enterica serovar Infantis recovered from surface waters in a major agricultural region for leafy greens in California
**Short Title:**	Plasmids in S. enterica serovar Infantis from surface waters
**Corresponding Author:**	Lisa Gorski<br>USDA Agricultural Research Service<br>Albany, CA UNITED STATES OF AMERICA
**Keywords:**	Salmonella; Infantis; plasmid; water; foodborne; pathogen
**Abstract:**	Non-typhoidal Salmonella enterica is a leading cause of gastrointestinal illnesses in the United States. Among the 2,600 different S. enterica serovars, Infantis has been significantly linked to human illnesses and is frequently recovered from broilers and chicken parts in the U.S. A key virulence determinant in serovar Infantis is the presence of the megaplasmid pESI, conferring multidrug resistance. To further characterize the virulence potential of this serovar, the present study identified the types of plasmids harbored by Infantis strains, recovered from surface waters adjacent to leafy greens farms in California. Sequencing analysis showed that each of the examined 12 Infantis strains had a large plasmid ranging in size from 78 kb to 125 kb. In addition, a second 4-kb plasmid was detected in two strains. Plasmid nucleotide queries did not identify the emerging megaplasmid pESI in the examined Infantis strains; however, the detected plasmids each had similarity to a plasmid sequence already cataloged in the nucleotide databases. Subsequent comparative analyses, based on gene presence or absence, divided the detected plasmids into five distinct clusters, and the phylogram revealed these Infantis plasmids were clustered based either on the plasmid conjugation system, IncI and IncF, or on the presence of plasmid phage genes. Assignment of the putative genes to functional categories revealed that the large plasmids contained genes implicated in cell cycle control and division, replication and recombination and defense mechanisms. Further analysis of the mobilome, including prophages and transposons, demonstrated the presence of genes implicated in the release of the bactericidal peptide microcin in the IncF plasmids and identified a Tn10 transposon conferring tetracycline resistance in one of the IncI1 plasmids. These findings indicated that the plasmids in the environmental S. enterica serovar Infantis strains from surface waters harbored a wide variety of genes associated with adaptation, survivability and antimicrobial resistance.
**Order of Authors:**	Beatriz Quiñones
	Bertram G. Lee
	Ashley Avilés Noriega
	Lisa Gorski
**Additional Information:**	
**Question**	**Response**
**Financial Disclosure**<br><br>Enter a financial disclosure statement that describes the sources of funding for the work included in this submission. Review the submission guidelines for detailed requirements. View published research articles from *PLOS ONE* for specific	Yes

Powered by Editorial Manager® and ProduXion Manager® from Aries Systems Corporation

examples.<br><br>This statement is required for submission and **will appear in the published article** if the submission is accepted. Please make sure it is accurate.<br><br>**Funded studies**<br>Enter a statement with the following details:<br>• Initials of the authors who received each award<br>• Grant numbers awarded to each author<br>• The full name of each funder<br>• URL of each funder website<br>• Did the sponsors or funders play any role in the study design, data collection and analysis, decision to publish, or preparation of the manuscript?<br><br>Did you receive funding for this work?	
Please add funding details.<br>as follow-up to "**Financial Disclosure**<br><br>Enter a financial disclosure statement that describes the sources of funding for the work included in this submission. Review the submission guidelines for detailed requirements. View published research articles from *PLOS ONE* for specific examples.<br><br>This statement is required for submission and **will appear in the published article** if the submission is accepted. Please make sure it is accurate.<br><br>**Funded studies**<br>Enter a statement with the following details:<br>• Initials of the authors who received each award<br>• Grant numbers awarded to each author<br>• The full name of each funder<br>• URL of each funder website<br>• Did the sponsors or funders play any role in the study design, data collection and analysis, decision to publish, or preparation of the manuscript?	This work supported by the United States Department of Agriculture, Agricultural Research Service, CRIS Project Numbers 2030-42000-052-00D and 2030-42000-055-00D. The funders had no role in study design, data collection and analysis, decision to publish, or preparation of the manuscript. Mention of trade names or commercial products in this publication is solely for the purpose of providing specific information and does not imply recommendation or endorsement by the USDA. USDA is an equal opportunity provider and employer.

Powered by Editorial Manager® and ProduXion Manager® from Aries Systems Corporation

Did you receive funding for this work?"	
Please select the country of your main research funder (please select carefully as in some cases this is used in fee calculation).<br>as follow-up to "**Financial Disclosure**<br><br>Enter a financial disclosure statement that describes the sources of funding for the work included in this submission. Review the submission guidelines for detailed requirements. View published research articles from *PLOS ONE* for specific examples.<br><br>This statement is required for submission and **will appear in the published article** if the submission is accepted. Please make sure it is accurate.<br><br>**Funded studies**<br>Enter a statement with the following details:<br>• Initials of the authors who received each award<br>• Grant numbers awarded to each author<br>• The full name of each funder<br>• URL of each funder website<br>• Did the sponsors or funders play any role in the study design, data collection and analysis, decision to publish, or preparation of the manuscript?<br><br>Did you receive funding for this work?"	UNITED STATES - US
**Competing Interests**<br><br>Use the instructions below to enter a competing interest statement for this submission. On behalf of all authors, disclose any competing interests that could be perceived to bias this work—acknowledging all financial support and any other relevant financial or non-financial competing interests.	The authors have declared that no competing interests exist.

This statement is required for submission and **will appear in the published article** if the submission is accepted. Please make sure it is accurate and that any funding sources listed in your Funding Information later in the submission form are also declared in your Financial Disclosure statement.<br><br>View published research articles from *PLOS ONE* for specific examples.<br><br>**NO authors have competing interests**<br><br>Enter: *The authors have declared that no competing interests exist.*<br><br>**Authors with competing interests**<br><br>Enter competing interest details beginning with this statement:<br><br>*I have read the journal's policy and the authors of this manuscript have the following competing interests: [insert competing interests here]*<br><br>* typeset	
**Ethics Statement**<br><br>Enter an ethics statement for this submission. This statement is required if the study involved:<br><br>• Human participants<br>• Human specimens or tissue<br>• Vertebrate animals or cephalopods<br>• Vertebrate embryos or tissues<br>• Field research<br><br>Write "N/A" if the submission does not require an ethics statement.<br><br>General guidance is provided below. Consult the submission guidelines for detailed instructions. **Make sure that all information entered here is included in the Methods section of the manuscript.**	N/A

**Format for specific study types**<br><br>**Human Subject Research (involving human participants and/or tissue)**<br>• Give the name of the institutional review board or ethics committee that approved the study<br>• Include the approval number and/or a statement indicating approval of this research<br>• Indicate the form of consent obtained (written/oral) or the reason that consent was not obtained (e.g. the data were analyzed anonymously)<br><br>**Animal Research (involving vertebrate animals, embryos or tissues)**<br>• Provide the name of the Institutional Animal Care and Use Committee (IACUC) or other relevant ethics board that reviewed the study protocol, and indicate whether they approved this research or granted a formal waiver of ethical approval<br>• Include an approval number if one was obtained<br>• If the study involved *non-human primates*, add *additional details* about animal welfare and steps taken to ameliorate suffering<br>• If anesthesia, euthanasia, or any kind of animal sacrifice is part of the study, include briefly which substances and/or methods were applied<br><br>**Field Research**<br><br>Include the following details if this study involves the collection of plant, animal, or other materials from a natural setting:<br>• Field permit number<br>• Name of the institution or relevant body that granted permission	
**Data Availability**<br><br>Authors are required to make all data underlying the findings described fully available, without restriction, and from the time of publication. PLOS allows rare exceptions to address legal and ethical concerns. See the PLOS Data Policy and FAQ for detailed information.	Yes - all data are fully available without restriction

A Data Availability Statement describing where the data can be found is required at submission. Your answers to this question constitute the Data Availability Statement and **will be published in the article**, if accepted.<br><br>**Important:** Stating 'data available on request from the author' is not sufficient. If your data are only available upon request, select 'No' for the first question and explain your exceptional situation in the text box.<br><br>Do the authors confirm that all data underlying the findings described in their manuscript are fully available without restriction?	
**Describe where the data may be found in full sentences. If you are copying our sample text, replace any instances of XXX with the appropriate details.**<br><br>• If the data are **held or will be held in a public repository**, include URLs, accession numbers or DOIs. If this information will only be available after acceptance, indicate this by ticking the box below. For example: *All XXX files are available from the XXX database (accession number(s) XXX, XXX.*).<br>• If the data are all contained **within the manuscript and/or Supporting Information files**, enter the following: *All relevant data are within the manuscript and its Supporting Information files.*<br>• If neither of these applies but you are able to provide **details of access elsewhere**, with or without limitations, please do so. For example:<br><br>*Data cannot be shared publicly because of [XXX]. Data are available from the XXX Institutional Data Access / Ethics Committee (contact via XXX) for researchers who meet the criteria for access to confidential data.*<br><br>*The data underlying the results presented in the study are available*	GenBank accession numbers PQ365737-PQ365750 with the BioProject accession number PRJNA1162552 in the National Center for Biotechnology Information (NCBI) BioProject database (https://www.ncbi.nlm.nih.gov/bioproject/).

from (include the name of the third party and contact information or URL).<br>• This text is appropriate if the data are owned by a third party and authors do not have permission to share the data.<br><br>* typeset	
Additional data availability information:	

Powered by Editorial Manager® and ProduXion Manager® from Aries Systems Corporation

Manuscript

Click here to access/download;Manuscript;2024.09.24_Manuscript_S.Infantis

RESEARCH ARTICLE

Plasmidome of *Salmonella enterica* serovar Infantis recovered from surface waters in a major agricultural region for leafy greens in California

Beatriz Quiñones, Bertram G. Lee, Ashley Avilés-Noriega, and Lisa Gorski*

Produce Safety and Microbiology Unit, Western Regional Research Center, Agricultural Research Service, U.S. Department of Agriculture, Albany, California, United States of America

*Corresponding author

Email: Lisa.Gorski@usda.gov (LG)

Short Title: Plasmids in *S. enterica* serovar Infantis from surface waters.

Abstract

Non-typhoidal *Salmonella enterica* is a leading cause of gastrointestinal illnesses in the United States. Among the 2,600 different *S. enterica* serovars, Infantis has been significantly linked to human illnesses and is frequently recovered from broilers and chicken parts in the U.S. A key virulence determinant in serovar Infantis is the presence of the megaplasmid pESI, conferring multidrug resistance. To further characterize the virulence potential of this serovar, the present study identified the types of plasmids harbored by Infantis strains, recovered from surface waters adjacent to leafy greens farms in California. Sequencing analysis showed that each of the examined 12 Infantis strains had a large plasmid ranging in size from 78 kb to 125 kb. In addition, a second 4-kb plasmid was detected in two strains. Plasmid nucleotide queries did not identify the emerging megaplasmid pESI in the examined Infantis strains; however, the detected plasmids each had similarity to a plasmid sequence already cataloged in the nucleotide databases. Subsequent comparative analyses, based on gene presence or absence, divided the detected plasmids into five distinct clusters, and the phylogram revealed these Infantis plasmids were clustered based either on the plasmid conjugation system, IncI and IncF, or on the presence of plasmid phage genes. Assignment of the putative genes to functional categories revealed that the large plasmids contained genes implicated in cell cycle control and division, replication and recombination and defense mechanisms. Further analysis of the mobilome, including prophages and transposons, demonstrated the presence of genes implicated in the release of the bactericidal peptide microcin in the IncF plasmids and identified a Tn10 transposon conferring tetracycline resistance in one of the IncI1 plasmids. These findings indicated that the plasmids in the environmental *S. enterica* serovar Infantis strains from surface waters harbored a wide variety of genes associated with adaptation, survivability and antimicrobial resistance.

Introduction

Non-typhoidal *Salmonella enterica* is one of the leading causes of foodborne illness in the United States [1-3]. Salmonellosis is a gastrointestinal illness of mild to moderate severity that can manifest approximately 72 h after exposure to infected food [3, 4]. The species *S. enterica* contains over 2,600 different serovars, which are differentiated by the combinations of the various somatic O and two phases of flagellar H antigens [5]. However, only about 100 serovars are implicated in most of the illness in humans [6, 7]. Since 2001, there has been a 167% increase in the incidence rate in infections caused by *S. enterica* serovar Infantis (*S.* Infantis) [6]. Among the serovars causing human illness, incidence of illness due to *S.* Infantis moved from the ninth most common in 1996 – 1998 to the sixth most common in 2019 [8] and since 2016, this serovar has been among the top five isolated from broilers and chicken parts [9]. One factor recognized in the emergence of serovar Infantis strains is the acquisition of a conjugative mega plasmid, termed as plasmid of emerging *S. enterica* Infantis (pESI) [10, 11]. First described in isolates from Israel in 2014, plasmid pESI was found to harbor multiple antimicrobial resistance genes, including an extended-spectrum-β-lactamase (ESBL) gene [10]. Since 2014, serovar Infantis isolates with this plasmid have been isolated worldwide, primarily from raw poultry; however, it is also present in wildlife and the environment [11, 12]. Multiple drug resistant strains of *S.* Infantis were reported as early as 1979 in isolates from wild birds in Florida [13].

Plasmids are important mediators of horizontal gene exchange, and may carry various types of resistance genes, including antibiotic resistance, metal resistance, and biocide resistance. They also can provide rapid adaptation to an organism during changing environmental conditions [14-16]. In the environment, strains of *Salmonella* are adept at cycling between host and non-host environments, including surface water, and can tolerate stress and nutrient depletion [17-22]. In

several surveys of an important produce-growing region along the Central California coast, *Salmonella* was routinely isolated, and some strains and subtypes showed indications of persistence and/or regular re-introduction into the environment [23-26]. In one survey of *Salmonella* from amphibians, reptiles, and surface waters, approximately 62% of the 106 *Salmonella* strains isolated were resistant to at least one antibiotic [25].

During a 5-year longitudinal survey (2011 – 2016) of surface waters of the Central California Coastal region, strains of *S.* Infantis were isolated from 34 samples collected during four of the five years from 13 of the 30 regular sampling sites [26]. Because of the increasing visibility of serovar Infantis strains, and the importance of plasmids in that emergence, the *S.* Infantis strains from that water survey were studied in depth for the occurrence of plasmids. The aim of this study was to determine plasmid carriage in the strains, and to characterize the types of plasmids among *S.* Infantis strains in this California water environment.

Materials and methods

DNA and Plasmid Extraction

The environmental *S.* Infantis strains used in this study were isolated from surface waters collected during the sampling years 2012 to 2016 [26] (S1 Table). Genomic DNA was extracted from single colonies grown in Trypticase soy broth + 0.6% yeast extract (TSYE, both ingredients from Difco, Franklin Lakes, Nj) using the Wizard Genomic DNA purification kit (Promega, Madison, WI) according to manufacturer's instructions except that the DNA was resuspended in Buffer EB (Qiagen, Hilden, Germany). Plasmids were extracted as described [27]. Briefly, *Salmonella* isolates were streaked on TSA agar and incubated overnight at 37 °C. Several large colonies were smeared in microcentrifuge tubes and resuspended in lysis buffer followed by

incubation at 55 °C for 60-90 minutes. Cell lysates were extracted with 100 µl phenol:chloroform:isoamyl alcohol (25:24:1) from which 50-70 µL of DNA were recovered. Samples were electrophoresed on a 0.7-0.8% agarose gel stained with GelRed in 1X Tris Borate EDTA buffer at 4 °C for 5 hours at 100V and visualized in a UV imager.

Library preparation and sequencing

Genomic DNA was prepared using the Wizard Genomic DNA Purification Kit (Promega, Madison, WI). The concentration and quality of the genomic DNA extracted from the recovered *Salmonella* strains was determined by using a NanoDrop ND-1000 spectrophotometer and a Qubit 4 fluorometer (Thermo Fisher Scientific,Waltham, MA USA). Long-read genome sequencing was performed using a MinION device (Oxford Nanopore Technologies, Oxford, UK). The sequencing libraries were prepared using a total of 200 ng DNA per *Salmonella* strain using a Rapid Barcoding Sequencing kit SQK-RBK-114.24 (Oxford Nanopore Technologies), following the manufacturer's workflow recommendations and procedures (https://community.nanoporetech.com/docs/prepare/library_prep_protocols/rapid-sequencing-gdna-barcoding-sqk-rbk114/v/rbk_9176_v114_revm_27nov2022/library-preparation?devices=minion (accessed on 1 March 2024). The prepared libraries were subsequently sequenced on FLO-MIN114 (version 10.4.1 active pore number ≥1100) flow cells (Oxford Nanopore Technologies) for a total of 48 h using MinKNOW software (v24.02.10) with the depletion option (adaptive sampling) using the reference genome from *S.* Infantis strain CVM N18S1246 (GenBank accession no. CP052777.1) to selectively increase the proportion of plasmid DNA to genomic DNA sequenced [28]. The sequencing reads were base called, trimmed, demultiplexed on super high accuracy mode, filtered on Phred Quality score 8, and saved in the

FASTQ format. Subsequently, the error correction and assembly of the sequencing reads were performed with Flye assembler version 2.9 [29] by using the recommended preset options for sequencing reads obtained with the Oxford Nanopore Technologies platform.

To resolve the plasmid sequences in the *S.* Infantis strains RM18512 and RM18281 into a single contig, the genomes for these strains were sequenced using the Illumina MiSeq System with 500-cycle v2 paired-end sequencing kits (Illumina, San Diego, CA, USA), following the manufacturer's instructions. DNA libraries were prepared and quantified using the KAPA Low-Throughput Library Preparation Kit and KAPA Library Quantification Kit (Roche, Kapa Biosystems, Wilmington, MA) with modifications as previously described [30]. Libraries were validated with the Agilent 2100 Bioanalyzer chips using the Agilent 12000 kit (Agilent, Santa Clara, CA). The combination of MiSeq and Nanopore sequencing reads were combined by using Unicycler software version v0.4.8 to obtain accurate and complete assemblies of short and long reads [31].

Sequence annotation and comparative analyses

The annotation of the plasmid sequences was performed by using RAST version 2.0 pipeline [10], were visualized using SnapGene software version 7.2 (GSL Biotech LLC, San Diego, CA), and were converted to a five-column tab-delimited annotation file using the GB2Sequin web application prior to submission to GenBank [32]. Confirmation of the Infantis serovar in the examined strains was performed with the SeqSero2 algorithm [33]. To identify the conjugation systems and mobilization families for each plasmid, the amino acid sequences were uploaded to the PlasmidFinder service to determine the mobilization family with MOBscan and to the Galaxy ToolShed site for identifying the conjugation system type with CONJscan [34-37]. To

screen the plasmid sequences, the Virulence Finder Database and the Comprehensive Antibiotic Resistance Database [38] were employed to search for genes related to virulence and antimicrobial resistance traits, respectively, and both databases were used in conjunction with the ABRicate tool, version 1.0.1, by selecting the parameter cutoffs of 90% coverage and 95% nucleotide identity [39]. Furthermore, the comparative analysis of the plasmid sequences was conducted using the Anvi'o platform [40], version 7, by selecting the pangenomics workflow system. The results obtained using the annotated genomic databases were subsequently visualized using the command entitled "Anvi-display-pan". Additionally, the data in the gene cluster summary file from the Anvi'o workflow system were used to analyze the distribution of functional clusters of orthologous gene categories in the examined *Salmonella* strains.

Data Availability

The annotated nucleotide sequences of the 14 plasmids in the examined *S. enterica* serovar Infantis strains are found under the GenBank accession numbers PQ365737-PQ365750 (Table 1) with the BioProject accession number PRJNA1162552 in the National Center for Biotechnology Information (NCBI) BioProject database (https://www.ncbi.nlm.nih.gov/bioproject/).

Results and Discussion

Comparative analysis of the non-pESI plasmids in the environmental *S. enterica* serovar Infantis strains

Initial sequencing analysis showed that 14 strains had a plasmid with sizes ranging from 78 kb to 125 kb (Table 1). The remaining 20 *S.* Infantis strains did not show bands in the plasmid screening (S1 Table). Twelve strains were selected for sequence analysis. BLASTN searches did not show sequence similarity to the emerging conjugative mega plasmid, pESI; however, each plasmid sequence had some similarity to a plasmid already cataloged in the database (Table 1). A comparative gene analysis was then conducted by employing the Anvi'o workflow to subsequently characterize the plasmid sequences in the examined *S.* Infantis strains. Phylogenetic analysis, based on gene presence or absence, in the *S.* Infantis strains divided the plasmid sequences into five distinct grouping (clusters) (Fig 1), and the phylogram revealed that the examined strains were clustered based on the plasmid conjugation system.

Fig 1. Comparative sequence analysis of the examined plasmids from *S. enterica* Infantis. The plasmid sequences identified *S. enterica* Infantis strains were annotated and further compared with the Anvi'o pangenome workflow and visualized with the "Anvi-display-pan" command.

Table 1. Plasmid characteristics in the examined *S. enterica* serovar Infantis strains from surface waters.

Cluster	*S*. Infantis strain ID	Plasmid name	Size (bp)	MOB family[a]	Conjugation system[b]	Number of genes[c]	Number of functional categories[d]	GenBank accession number	GenBank accession number of related plasmid (plasmid name)
I	RM16752	pRM16752-2	4,074	none	none	4	2	PQ365738	CP050752.1 (plasmid pST46-2)
	RM17050	pRM17050-2	4,074	none	none	4	2	PQ365739	CP050752.1 (plasmid pST46-2)
II	RM17050	pRM17050-1	78,346	MOB$_P$	IncI1	85	19	PQ365740	CP136836.1 (plasmid pK2721-1)
	RM18148	pRM18148	91441	MOB$_P$	IncI1	103	25	PQ365744	OP378609 (plasmid pWP2_ecoli)
	RM18410	pRM18410	88,744	MOB$_P$	IncI1	102	25	PQ365746	LC567088 (plasmid pWP2_ecoli)
	RM16752	pRM16752-1	84,544	MOB$_P$	IncI1	98	21	PQ365737	CP136836.1 (plasmid pK2721-1)
	RM18147	pRM18147	84,109	MOB$_P$	IncI1	99	23	PQ365743	AP021916 (plasmid pWP2_ecoli)
III	RM21154	pRM21154	90,009	None	None	119	24	PQ365750	CP111090 (plasmid p2013K-1747)
	RM17884	pRM17884	92,091	None	None	115	23	PQ365741	CP111090 (plasmid p2013K-1747)
	RM17885	pRM17885	87,819	None	None	121	25	PQ365742	CP111090 (plasmid p2013K-1747)
	RM21071	pRM21071	87,783	None	None	113	23	PQ365748	CP111090 (plasmid p2013K-1747)
IV	RM21082	pRM21082	91,487	MOB$_F$	IncF	108	40	PQ365749	CP077697 (plasmid pCFSAN058598)
V	RM18512	pRM18512	116,460	MOB$_F$	IncF	142	58	PQ365747	CP022660 (plasmid pRM11060-2)
	RM18281	pRM18281	126,613	MOB$_F$	IncF	158	58	PQ365745	CP022660 (plasmid pRM11060-2)

[a]The mobilization (MOB) family was determined with MOBscan by uploading the amino acid sequences to the PlasmidFinder service [35, 37].

[b]The conjugation system was determined with CONJscan by uploading the amino acid sequences to the Galaxy ToolShed site [34, 36].

[c]Number of genes represents coding sequences as identified using RAST version 2.0 pipeline [10].

[d]Number of functional categories represents the clusters of orthologous genes, based on the gene cluster summary file with the Anvi'o workflow [40].

Moreover, further analysis of the contigs revealed a small multicopy 4 kb-plasmid in strains RM17050 and RM16752, which was assigned to cluster I (Fig 1 and Table 1). The IncI1-based conjugation system, belonging to the MOB_P family, was identified in cluster II plasmids pRM16752-1, pRM17050-1, pRM18147, pRM18148 and pRM18410. By contrast, the IncF-based conjugation system, belonging to the MOB_F family, was detected in plasmids pRM18512, pRM18281 and pRM21082. However, plasmid pRM21082, assigned to cluster IV, was only identified in strain RM21082 and was found to be 3000 bp smaller than the plasmids pRM18512 and pRM18281, which were assigned to cluster V. Moreover, plasmids pRM17884, pRM17885, pRM21071 and pRM21154, assigned to cluster III, did not contain a conjugation system (Table 1); however, further analysis of the sequences with PHASTEST, a web server designed to identify prophage sequences [41], revealed that the cluster III plasmids resembled a phage plasmid, which was documented as a plasmid type with a potential for both horizontal and vertical transfers [42].

Additional sequence comparison of the Infantis plasmids with those deposited in the databases indicated that the plasmids from this study showed significant sequence similarity to plasmids previously identified both locally and across the globe. In particular, the cluster I plasmids pRM16752-2 and pRM17050-2 each shared 100% identity (99% coverage) to plasmid pST46-2 (Table 1); the small plasmid pST46-2 was sequenced from a multidrug resistant *S. enterica* serovar Typhimurium strain 46 isolated from a clinical sample in China [43]. The cluster III phage plasmids showed the closest sequence similarity (99% identity; 88-99% coverage) to a plasmid found in the clinical strain, *S. enterica* 2013K-1747 (Table 1). Although there is limited published information about location, transmission vehicle and illness caused by strain 2013K-1747, the genome sequence was included as part of a larger study of genomic typing schemes for *Salmonella* [44]. Subsequent genome sequence analysis, using SeqSero2

algorithm [33], revealed strain 2013K-1747 to be a *S. enterica* serovar Adalaide. The appearance of very similar phage plasmids in two serovars, Infantis and Adalaide, indicate movement of this type of plasmid among *Salmonella*. Moreover, the large cluster V IncF plasmids, pRM18512 and pRM18281, showed sequence similarity to plasmid pRM11060-2 (100% identity; 40% coverage), previously identified in *S. enterica* serotype 6,8:d:− from surface waters collected in Central California in 2009 [45]. Comparative analysis indicated plasmid pRM11060-2 contained an additional 154-kb fragment encoding a prophage and hemolysin [45]. The *S.* Infantis strains RM18512 and RM18281 were also isolated from water samples collected in 2013 – 2014 in the same agricultural region [26]. These observations suggested that some genes in the IncF plasmid, detected in both serovars, Infantis and 6,8:d:−, appeared to be prevalent in this agricultural region among different strains and serotypes of *Salmonella*.

As further demonstrated in the comparative analysis with the Anvi'o workflow (Fig 1), various homogeneity indexes were determined to measure the variation and frequency of their occurrence in the detected plasmid genes with an index of 1 being the highest value indicating no variation. In this study, the average homogeneity index value was determined to be 0.95 to 0.96 for all plasmid sequences combined. Subsequent measurement of the geometric and functional homogeneity indexes was performed. In particular, the geometric homogeneity index determined the similarity between gene variations based on how many gaps exist between the different sequences in the gene. The functional homogeneity index measured the similarity between gene variations based on how many differences exist between aligned sequences of the gene variations. The results obtained in this study identified notable genes, linked to plasmid stability, segregation and replication [46-48], with lower geometric homogeneity such as the DNA methylase subunit (HsdM) in the IncI1 plasmids with an index of 0.64, the chromosome segregation protein (Spo0J) in the IncF plasmids with an index of 0.65, and the DNA polymerase

(DinB) with an index of 0.64. Additionally, other identified genes, associated with plasmid transferability and replication [49-51], with lower functional homogeneity were the single-stranded DNA-binding protein (Ssb) among the IncF and IncI1 plasmids with an index of 0.80, the type IV secretory pathway component (VirB4) among the IncF plasmids with an index of 0.71, and the SOS-response transcriptional repressor (LexA) among the IncF and IncI1 plasmids with an index of 0.81. The high degree of variability in these genes with lower geometric and functional homogeneity scores could potentially have an effect on important traits related to plasmid copy number, stability and distribution within a bacterial population and subsequently influence the bacterial host virulence, adaptation and fitness to environments [15, 16].

Characterization of the functional gene categories in the examined plasmids from *S. enterica* serovar Infantis strains

Further analysis of the detected genes in the Infantis plasmids revealed that on average 25% of the genes in all of the examined plasmids were assigned to functional categories (Table 1). In particular, the two small 4 kb-plasmids in cluster I had the highest proportion of assigned categories. The cluster II and cluster III plasmids with an average plasmid size of 85.4 kb to 89.4 kb had about 20-23% of detected genes with an assigned function. The largest plasmids in clusters IV and V with an average size of 91.5 kb and 121 kb, respectively, were found to have a higher proportion of the genes (38%) assigned to functional categories (Table 1). By conducting a subsequent analysis with the Anvi'o workflow [40], the presence of putative genes for strains belonging to each grouping was identified, and the genes assigned to specific functional categories were further examined (Fig 2). All the large plasmids (>78 Kb) contained at least 4% of genes in the functional for cell cycle control and cell division (D category), replication and recombination (L category) as well as defense mechanisms and transcription (V and K

categories, respectively). Subsequent analysis of cell cycle control and division functional category revealed the presence in the large plasmids of parA and paB-like genes, which have been implicated in the ParABS DNA partitioning system, which was described originally in low copy number plasmids [52, 53].

Fig 2. Functional gene categories identified in the *S. enterica* Infantis plasmids. The assignment to functional clusters of orthologous genes categories was determined from the gene cluster summary file using the Anvi'o workflow for the examined *S.* Infantis plasmids, grouped in various clusters.

Examination of genes involved in replication, recombination and repair demonstrated the large plasmids contained a site-specific recombinase *xerD* like gene [54]. The phage-containing plasmids in cluster III and IncF plasmids in cluster II (p91487, p116460, and p126613) contained an additional site-specific recombinase like gene resembling *pinE*. These phage-containing plasmids also harbor a DNA-methylase like gene and a gene resembling a DNA recombination-dependent growth factor RdgC. The remaining plasmids had a different DNA-methylase *yhdj* identified, as well as genes supporting DNA repair.

To characterize genes associated with defense mechanisms, cluster III plasmids were found to contain *higA* and *higB* like genes, encoding the HigAB toxin antitoxin module for plasmid maintenance and possible virulence modification [55]. Additionally, the *relBE* family of Type II toxin-antitoxin system found in IncR1 plasmids may be involved in stress response [56, 57]. The IncI1 plasmids, including pRM18148, contained the *ardA* antirestriction gene which may allow the spread of virulence factors contained in the plasmid (McMahon 2009), while the main IncF plasmids contained type I restriction system as well as an *emrAB* coding for an efflux

pump system associated with multidrug and chromate resistance [58, 59]. Plasmid pRM21154 contained a dCTP deaminase (Dcd), an enzyme required for normal growth in gram negative bacteria since it is required for the synthesis of deoxyribonucleotides [60].

On the subsequent analysis of genes involved in transcription, all large plasmids contained a form of a SOS-response transcriptional repressor LexA. In particular, plasmids pRM18512 and pRM18281 contained a predicted transcriptional regulator with a different ribbon-helix-helix (RHH_1) domain and predicted DNA-binding transcriptional regulator. Plasmid pRM21082 contained a predicted RNA polymerase-binding transcription factor DksA, implicated in the antioxidative stress response with a role in *Salmonella* pathogenesis [61], and a putative transcriptional regulator GlxA. Most IncI1 plasmids, with the exception of pRM18148, contained a predicted putA1 ribbon-helix-helix transcription regulator while plasmid pRM18148 alternately contained predicted DNA-binding proteins in the AraC and AcrA family.

Analysis of the mobilome functional category X, including prophages and transposons, showed that cluster III plasmids contained genes involved in regulation and structure of the phage while IncF plasmids pRM18512 and pRM18281 contained a transposase system that encoded microcin V (*mccV*), a low molecular weight peptide with bactericidal effects to species within the genera *Klebsiella*, *Escherichia*, *Salmonella*, and *Shigella* [62]. Interestingly, these two IncF plasmids, pRM18512 and pRM18281, also contained a predicted arabinose efflux permease *araJ* like gene, which was classified in the carbohydrate transport functional category G. Previous findings have implicated arabinose as an inducer of biofilm production in *S.* Typhimurium [63]. Additionally, both of these plasmids also harbored the *mefA*, encoding a macrolide efflux protein A [64]. MefA is a member of the major facilitator superfamily and has been implicated in the uptake of nutrients and extrusion of harmful compounds such as antibiotics and heavy metals [64, 65].

The identification of genes in the cluster III phage plasmids with a role in the biogenesis of the cell wall and membrane (category M) revealed genes with a lytic trans-glycosylase *lysM* domain, which has been proposed to be involved in routine maintenance of cell walls, resistance against cell-wall acting antibodies, cell wall hydrolases, and host invasion [66]. Moreover, the analysis of functional genes implicated in signal transduction mechanisms (category T) demonstrated that cluster III plasmids also contained *cpdA* gene coding for 3',5'-cyclic AMP phosphodiesterase, implicated in cyclic 3',5'-AMP production as a global virulence regulator or for the regulation of cAMP under growth conditions with limited glucose [67]. The two large IncF plasmids, pRM18512 and pRM18281, contained an sRNA binding gene *proQ*, associated with the activation of *Salmonella* virulence genes [68]. On the analysis of cell motility, the IncI1 plasmids contained the effector genes, *pilK-pilV* associated with the type II secretion system, including a pseudopilin, resembling a flagellum. In other *Salmonella* isolates, the *pil* genes on the IncI1 R64 plasmid encode the Type IVb pilus for conjugation [69]. The genes in *pilK-pilV* are expressed in *Salmonella* Typhi and are thought to play a role in the virulence of that pathogen. Mutants in *pilS*, the major pilin subunit, had reduced adhesion and invasion of human epithelial gastrointestinal cells *in vitro* [70, 71]. Whether these genes affect the potential virulence of the *S.* Infantis strains in this study would be an avenue for future study. Plasmid pRM21082 harbored a pilus assembly gene *fimC* encoding for a chaperone and was the only gene identified in the functional category assigned to extracellular structures (category W).

Tetracycline resistance determinants are present in the *S.* Infantis plasmid pRM18148

By using a validated database to screen for antimicrobial resistance genes, the analysis revealed that only of the examined Infantis plasmids, the IncI plasmid pRM18148 had genes

conferring tetracycline resistance (Fig 3). Phenotypically, strain RM18148 was tetracycline resistant (data not shown). Subsequent sequence alignments revealed that the *S.* Infantis plasmid pRM18148 showed high sequence similarity (99%) to plasmid pN18S0935-1 in *S.* serovar Kentucky strain N18S0935 and to plasmid pN17S166 recovered from *S. enterica* serovar 4,[5],12:i:− strain CVM N17S166 [72]. One difference identified in the Infantis plasmid pRM18148 was the presence of a putative adenosylcobinamide-phosphate synthase (*cbiB*) gene (Fig 3), a region with a potential role in conjugation and coenzyme B_{12} biosynthesis in serovar Typhimurium, respectively [73]. Further analysis with the TnCentral web portal [74] of the region conferring tetracycline resistance in the Infantis plasmid pRM18148 indicated that these antimicrobial resistance genes were contained within a partial sequence of the Tn10 transposon (Fig 3), a composite mobile element which was also previously found in an unnamed plasmid identified in *S. enterica* serovar Heidelberg strain FDAARGOS_319 (Accession No. CP027411)[75, 76]. Analysis of the coding sequences in the Infantis plasmid pRM18148 confirmed the presence of the TetB efflux pump, flanked by the tetracycline resistance transcriptional repressor TetR, the tetracyline resistance-associated transcriptional repressor TetC, and the multidrug resistant efflux pump AcrAB transcriptional activator RobA.

Fig 3. Schematic diagram of *S.* Infantis plasmid pRM18148 conferring tetracycline resistance. The RAST annotated nucleotide sequences for plasmid pRM18148 were uploaded to SnapGene software, version 7.2 (GSL Biotech LLC, San Diego, CA) to generate the plasmid map.

Other notable features identified in the Infantis plasmid pRM18148 included the SOS-inhibitors PsiA and PsiB, involved in maintaining the integrity of the plasmid DNA and host

genome as well as the plasmid-encoded methyltransferase YhdJ and the DNA methylase HsdM with a potential role in protecting plasmid DNA from restriction modification systems, as mechanisms that may aid in evading the defense of the invaded host [77, 78]. Additionally, plasmid pRM18148 was found to harbor a shufflon-specific DNA recombinase (Fig 3), a multi-inversion region in IncI1 plasmids and affecting the formation of the plasmid conjugation apparatus [79] as well as the gene encoding the antirestriction protein ArdA with a functional role in bacterial conjugation to allow an unmodified plasmid to evade restriction modification system in the recipient host [80]. On the identification of the conjugal apparatus, plasmid pRM18148 contained the *traABC* region, which encode regulators required for the expression of the IncI1-transfer associated genes [79], and this *tra* region was found upstream of the *pil* gene cluster, required for the formation of the type IV pilus. In addition, plasmid pRM18148 was found to harbor the relaxase protein NikB, which is required for the formation of the relaxosome to initiate the conjugal transfer of the plasmid DNA through the secretion system [79].

Conclusions

Plasmids are vectors for transferring resistance, virulence, and adaptation genes in bacteria [14-16]. The comparative sequence analysis of the plasmids among the *S.* Infantis strains, isolated from a major agricultural region Central California [26], grouped some of the plasmids into various clusters based on previously described conjugation groups IncI1 and IncF, and indicated the ability of these plasmid types to move among the microbial population. In particular, IncI1 plasmids are a common plasmid type among the *Enterobacteriaceae* and have been isolated from foods and human clinical cases. Because of their potential to carry antibiotic resistance and virulence genes, they are a cause of public health concern [79]. Studies with *Escherichia coli* and *Salmonella* 4,5,12:i:− strains showed that carriage of the IncI1 plasmid

carried no measurable fitness cost on the bacteria in terms of association and invasion *in vitro* [79, 81]. IncF plasmids are also known for transferring antibiotic resistance determinants. The IncF family may contain SpvB, an ADP-ribosylating toxin, which allows it to cause systemic infection in humans. To date, the SvpB-IncF plasmid has been found in nine different *Salmonella* serotypes, not including Infantis [82-84]. The pESI plasmid is a member of the IncF1B family [11]. Interestingly, the similarity of the Infantis IncF plasmids to another plasmid (pRM11060-2), previously described from a different *Salmonella* with the serotype 6,8:d:− [45], indicates that the plasmids in this study remain in the surveyed agricultural region or are regularly re-introduced, and move between serovars of *Salmonella*. Moreover, the present study also documented phage plasmids in the Infantis strains. Phage plasmids have been described previously in *Salmonella* [42, 85, 86], and this type of mobilizable genetic elements are capable of conferring extended-spectrum β-lactamase resistance in *S. enterica* serovar Typhi and other bacteria [42, 87].

Analysis of the homogeneity indexes indicated that a high degree of gene variability was identified in relevant traits related to plasmid segregation, conjugation, replication, and stability. Variability in these plasmid genes could potentially have an effect on the ability of these plasmids to move determinants among different genera of bacteria or different hosts within a genera in the environment [15, 84]. Although no virulence factors were detected according to the output provided by Virulence Finder Database, analysis of the functional gene categories in the Infantis plasmids identified several pathogenesis determinants conferring *Salmonella* an ability to adhere, invade as well as colonize and survive in the host mammalian cells. Additionally, the use of the Comprehensive Antibiotic Resistance Database revealed that only plasmid pRM18148 carried an antibiotic resistance marker. Sequence analysis of the IncI1 plasmid pRM18148 identified markers conferring resistance to tetracycline, and these determinants were carried on a

Tn10 transposon, a well-characterized composite element with the ability to move between other plasmids and host genomes [76]. Phenotypic resistance to tetracycline in the Infantis strain RM18148 was corroborated based on results obtained by performing the disk diffusion assay (data not shown). Tetracylines are widely used agents in agricultural settings [88], and resistance to these agents has been one of the most commonly identified in the plasmids among *S. enterica* isolates from food animals in the United States [89], highlighting the need for better monitoring on the use of these agents in agriculture. In summary, the present study employed high-resolution sequencing to characterize mobilizable plasmids, belonging to different incompatibility groups, in *S. enterica* serovar Infantis strains, recovered from surface waters adjacent to leafy greens farms in California. This analysis led to the identification of pathogenesis and antimicrobial resistance determinants that can contribute to the virulence potential of the *S*. Infantis strains from an important agricultural region.

Acknowledgements

The authors thank Dr. Dayna Harhay (USDA-ARS, U.S. Meat Animal Research Center, Clay Center, NE) for providing technical advice and *E. coli* strains harboring control plasmids.

References

1. Dewey-Mattia D, Manikonda K, Hall AJ, Wise ME, Crowe SJ. Surveillance for foodborne disease outbreaks - United States, 2009-2015. Morbidity and Mortality Weekly Report Surveillance Summaries. 2018;67(10):1-11. doi: 10.15585/mmwr.ss6710a1.
2. U.S. Centers for Disease Control. FoodNet Fast pathogen surveillance 2023 [cited 2023 5 September]. Available from: https://wwwn.cdc.gov/foodnetfast/.

3. Scallan Walter EJ, Griffin PM, Bruce BB, Hoekstra RM. Estimating the number of illnesses caused by agents transmitted commonly through food: A scoping review. Foodborne Pathog Dis. 2021;18(12):841-858. Epub 2021/09/17. doi: 10.1089/fpd.2021.0038.

4. U.S. Centers for Disease Control and Prevention. *Salmonella* 2024 [cited 2024 10 July]. Available from: https://www.cdc.gov/salmonella/index.html.

5. Grimont PAD, Weill F-X. Antigenic formulae of the *Salmonella* serovars, 9th edition. Paris, France: Pasteur Institute: World Health Organization Collaborating Centre for Reference and Research on *Salmonella*; 2007.

6. U.S. Centers for Disease Control and Prevention. National enteric disease surveillance: *Salmonella* annual report, 2016 2018 [cited 2024 8 July]. Available from: https://www.cdc.gov/nationalsurveillance/pdfs/2016-Salmonella-report-508.pdf.

7. European Food Safety Authority. The European Union One Health 2021 Zoonoses Report. EFSA Journal. 2022;20(12):e07666. doi: 10.2903/j.efsa.2022.7666.

8. Tack DM, Ray L, Griffin PM, Cieslak PR, Dunn J, Rissman T, et al. Preliminary incidence and trends of infections with pathogens transmitted commonly through food — Foodborne Diseases Active Surveillance Network, 10 U.S. sites, 2016 - 2019. MMWR Morb Mortal Wkly Rep. 2020;69(17):509-514. doi: 10.15585/mmwr.mm6917a1.

9. U.S. Department of Agriculture Food Safety Inspection Service. Quarterly sampling reports on *Salmonella* and *Campylobacter* 2024 [cited 2024 9 July]. Available from: https://www.fsis.usda.gov/science-data/data-sets-visualizations/microbiology/microbiological-testing-program-rte-meat-and-7.

10. Aviv G, Tsyba K, Steck N, Salmon-Divon M, Cornelius A, Rahav G, et al. A unique megaplasmid contributes to stress tolerance and pathogenicity of an emergent *Salmonella*

enterica serovar Infantis strain. Environmental Microbiology. 2014;16(4):977-994. doi: 10.1111/1462-2920.12351.

11. Alvarez DM, Barrón-Montenegro R, Conejeros J, Rivera D, Undurraga EA, Moreno-Switt AI. A review of the global emergence of multidrug-resistant *Salmonella enterica* subsp. *enterica* serovar Infantis. International Journal of Food Microbiology. 2023;403:110297. doi: 10.1016/j.ijfoodmicro.2023.110297.
12. McMillan EA, Weinroth MD, Frye JG. Incresed prevalence of *Salmonella* Infantis isolated from raw chicken and turkey products in the United States is due to a single clonal lineage carrying the pESI plasmid. Microorganisms. 2022;10:1478. doi: 10.3390/microorganisms10071478.
13. White FH, Forrester DJ. Antimicrobial resistant *Salmonella* spp. isolated from double-crested cormorants (*Phalocrocorax auritus*) and common loons (*Gavia immer*) in Florida. J Wildlife Dis. 1979;15(2):235-237. doi: 10.7589/0090-3558-15.2.235.
14. Smalla K, Jechalke S, Top EM. Plasmid detection, characterization, and ecology. Microbiology Spectrum. 2015;3(1):PLAS-0038-2014. doi: 10.1128/microbiolspec.PLAS-0038-2014.
15. Rodríguez-Beltrán J, DelaFuente J, León-Sampedro R, MacLean RC, San Millán Á. Beyond horizontal gene transfer: The role of plasmids in bacterial evolution. Nat Rev Microbiol. 2021;19(6):347-359. doi: 10.1038/s41579-020-00497-1.
16. Wein T, Dagan T. Plasmid evolution. Curr Biol. 2020;30(19):R1158-R1163. Epub 2020/10/07. doi: 10.1016/j.cub.2020.07.003.
17. Winfield MD, Groisman EA. Role of nonhost environments in the lifestyles of *Salmonella* and *Escherichia coli*. Appl Environ Microbiol. 2003;69(7):3687-3694. doi: 10.1128/AEM.69.7.3687-3694.2003.

18. Liu H, Whitehouse CA, Li B. Presence and persistence of *Salmonella* in water: the impact on microbial quality of water and food safety. Frontiers in Public Health. 2018;6:159. doi: 10.3389/fpubh.2018.00159.

19. Topalcengiz Z, McEgan R, Danyluk MD. Fate of *Salmonella* in Central Florida surface waters and evaluation of EPA worst case water as a standard medium. Journal of Food Protection. 2019;82(6):916-925. doi: 10.4315/0362-028X.JFP-18-331.

20. Thomason BM, Biddle JW, Cherry WB. Detection of Salmonellae in the environment. Appl Microbiol. 1975;30(5):764-767. doi: 10.1128/am.30.5.764-767.1975.

21. Parker CT, Huynh S, Quiñones B, Harris LJ, Mandrell RE. Comparison of genotypes of *Salmonella enterica* serovar Enteritidis phage type 30 and 9c strains isolated during three outbreaks associated with raw almonds. Appl Environ Microbiol. 2010;76(11):3723-3731.

22. Fatica MK, Schneider KR. *Salmonella* and produce: survival in the plant environment and implications in food safety. Virulence. 2011;2:573-579. doi: 10.4161/viru.2.6.17880.

23. Cooley MB, Quiñones B, Oryang D, Mandrell RE, Gorski L. Prevalence of Shiga toxin-producing *Escherichia coli*, *Salmonella enterica*, and *Listeria monocytogenes* at public access watershed sites in a California Central Coast agricultural region. Front Cell Infect Microbiol. 2014;4:30. doi: 10.3389/fcimb.2014.00030.

24. Gorski L, Parker CT, Liang A, Cooley MB, Jay-Russell MT, Gordus A, et al. Prevalence, distribution, and diversity of *Salmonella enterica* in a major produce region of California. Appl Environ Microbiol. 2011;77(8):2734-2748. doi: 10.1128/AEM.02321-10.

25. Gorski L, Jay-Russell MT, Liang AS, Walker S, Bengson Y, Govoni J, et al. Diversity of pulsed field gel electrophoresis pulsotypes, serovars and antibiotic resistance among *Salmonella* isolates from wild amphibians and reptiles in the California Central Coast. Foodborne Pathogens and Disease. 2013;10:540-548. doi: 10.1089/fpd.2012.1372.

26. Gorski L, Liang AS, Walker S, Carychao D, Aviles Noriega A, Mandrell RE, et al. *Salmonella enterica* serovar diversity, distribution, and prevalence in public-access waters from a Central California coastal leafy green-growing region from 2011 to 2016. Appl Environ Microbiol. 2022;88:e01834-21. doi: 10.1128/AEM.01834-21.
27. Kado CI, Liu ST. Rapid procedure for detection and isolation of large and small plasmids. J Bacteriol. 1981;145(3):1365-1373. doi: 10.1128/jb.145.3.1365-1373.1981.
28. Ulrich JU, Epping L, Pilz T, Walther B, Stingl K, Semmler T, et al. Nanopore adaptive sampling effectively enriches bacterial plasmids. mSystems. 2024;9(3):e0094523. Epub 2024/02/20. doi: 10.1128/msystems.00945-23.
29. Kolmogorov M, Yuan J, Lin Y, Pevzner PA. Assembly of long, error-prone reads using repeat graphs. Nat Biotechnol. 2019;37(5):540-546. doi: 10.1038/s41587-019-0072-8.
30. Parker CT, Huynh S, Alexander A, Oliver AS, Cooper KK. Genomic characterization of *Salmonella typhimurium* DT104 Strains associated with cattle and beef products. Pathogens. 2021;10(5):529. doi: 10.3390/pathogens10050529.
31. Wick RR, Judd LM, Gorrie CL, Holt KE. Unicycler: Resolving bacterial genome assemblies from short and long sequencing reads. PLoS Comput Biol. 2017;13(6):e1005595. doi: 10.1371/journal.pcbi.1005595.
32. Lehwark P, Greiner S. GB2sequin - A file converter preparing custom GenBank files for database submission. Genomics. 2019;111(4):759-761. doi: 10.1016/j.ygeno.2018.05.003.
33. Zhang S, den Bakker Hendrik C, Li S, Chen J, Dinsmore Blake A, Lane C, et al. SeqSero2: Rapid and improved *Salmonella* serotype determination using whole-genome sequencing data. Appl Environ Microbiol. 2019;85(23):e01746-19. doi: 10.1128/AEM.01746-19.

34. Blankenberg D, Von Kuster G, Bouvier E, Baker D, Afgan E, Stoler N, et al. Dissemination of scientific software with Galaxy ToolShed. Genome Biol. 2014;15(2):403. doi: 10.1186/gb4161.

35. Carattoli A, Hasman H. PlasmidFinder and In Silico pMLST: Identification and Typing of Plasmid Replicons in Whole-Genome Sequencing (WGS). In: de la Cruz F, editor. Horizontal Gene Transfer: Methods and Protocols. New York, NY: Springer US; 2020. p. 285-294.

36. Cury J, Abby SS, Doppelt-Azeroual O, Néron B, Rocha EPC. Identifying conjugative plasmids and integrative conjugative elements with CONJscan. In: de la Cruz F, editor. Horizontal Gene Transfer: Methods and Protocols. New York, NY: Springer US; 2020. p. 265-283.

37. Garcillán-Barcia MP, Redondo-Salvo S, Vielva L, de la Cruz F. MOBscan: Automated annotation of MOB relaxases. In: de la Cruz F, editor. Horizontal Gene Transfer: Methods and Protocols. New York, NY: Springer US; 2020. p. 295-308.

38. Alcock BP, Huynh W, Chalil R, Smith KW, Raphenya Amogelang R, Wlodarski MA, et al. CARD 2023: expanded curation, support for machine learning, and resistome prediction at the Comprehensive Antibiotic Resistance Database. Nucleic Acids Res. 2023;51(D1):D690-D699. doi: 10.1093/nar/gkac920.

39. Seemann T. ABRicate 2019 [cited 2024 1 May]. Available from: https://github.com/tseemann/abricate.

40. Eren AM, Kiefl E, Shaiber A, Veseli I, Miller SE, Schechter MS, et al. Community-led, integrated, reproducible multi-omics with anvi'o. Nat Microbiol. 2021;6(1):3-6. doi: 10.1038/s41564-020-00834-3.

41. Wishart DS, Han S, Saha S, Oler E, Peters H, Grant Jason R, et al. PHASTEST: faster than PHASTER, better than PHAST. Nucleic Acids Res. 2023;51(W1):W443-W450. doi: 10.1093/nar/gkad382.

42. Pfeifer E, Rocha EPC. Phage-plasmids promote recombination and emergence of phages and plasmids. Nat Commun. 2024;15(1):1545. doi: 10.1038/s41467-024-45757-3.

43. Chen Z, Kuang D, Xu X, González-Escalona N, Erickson DL, Brown E, et al. Genomic analyses of multidrug-resistant *Salmonella* Indiana, Typhimurium, and Enteritidis isolates using MinION and MiSeq sequencing technologies. PLoS One. 2020;15(7):e0235641. Epub 2020/07/03. doi: 10.1371/journal.pone.0235641.

44. Leeper MM, Tolar BM, Griswold T, Vidyaprakash E, Hise KB, Williams GM, et al. Evaluation of whole and core genome multilocus sequence typing allele schemes for *Salmonella enterica* outbreak detection in a national surveillance network, PulseNet USA. Front Microbiol. 2023;14:1254777. Epub 2023/10/09. doi: 10.3389/fmicb.2023.1254777.

45. Gorski L, Huynh S, Cooper K, K., Parker C, T. Complete genomic sequences of two *Salmonella enterica* subsp. *enterica* serogroup C2 (O:6,8) strains from Central California. Genome Announc. 2017;5(46):e01234-17. doi: 10.1128/genomea.01234-17.

46. Gao Q, Lu S, Wang Y, He L, Wang M, Jia R, et al. Bacterial DNA methyltransferase: A key to the epigenetic world with lessons learned from proteobacteria. Front Microbiol. 2023;14:1129437. doi: 10.3389/fmicb.2023.1129437.

47. Lee PS, Lin DC, Moriya S, Grossman AD. Effects of the chromosome partitioning protein Spo0J (ParB) on *oriC* positioning and replication initiation in *Bacillus subtilis*. J Bacteriol. 2003;185(4):1326-1337. Epub 2003/02/04. doi: 10.1128/jb.185.4.1326-1337.2003.

48. Wagner J, Gruz P, Kim S-R, Yamada M, Matsui K, Fuchs RPP, et al. The *dinB* gene encodes a novel *E. coli* DNA polymerase, DNA Pol IV, involved in mutagenesis. Mol Cell. 1999;4(2):281-286. doi: 10.1016/S1097-2765(00)80376-7.

49. Bianco PR. The mechanism of action of the SSB interactome reveals it is the first OB-fold family of genome guardians in prokaryotes. Protein Sci. 2021;30(9):1757-1775. Epub 2021/06/06. doi: 10.1002/pro.4140.

50. Fornelos N, Browning DF, Butala M. The use and abuse of LexA by mobile genetic elements. Trends Microbiol. 2016;24(5):391-401. doi: 10.1016/j.tim.2016.02.009.

51. Gokulan K, Khare S, Rooney AW, Han J, Lynne AM, Foley SL. Impact of plasmids, including those encodingVirB4/D4 type IV secretion systems, on *Salmonella enterica* serovar Heidelberg virulence in macrophages and epithelial cells. PLoS One. 2013;8(10):e77866. Epub 2013/10/08. doi: 10.1371/journal.pone.0077866.

52. Badrinarayanan A, Le TBK, Laub MT. Rapid pairing and resegregation of distant homologous loci enables double-strand break repair in bacteria. J Cell Biol. 2015;210(3):385-400. doi: 10.1083/jcb.201505019.

53. Köhler R, Kaganovitch E, Murray SM. High-throughput imaging and quantitative analysis uncovers the nature of plasmid positioning by ParABS. eLife. 2022;11:e78743. doi: 10.7554/eLife.78743.

54. Midonet C, Barre F-X. Xer site-specific recombination: Promoting vertical and horizontal transmission of genetic information. Microbiology Spectrum. 2014;2(6):MDNA3-0056-2014. doi: 10.1128/microbiolspec.mdna3-0056-2014.

55. Wood TL, Wood TK. The HigB/HigA toxin/antitoxin system of *Pseudomonas aeruginosa* influences the virulence factors pyochelin, pyocyanin, and biofilm formation. Microbiologyopen. 2016;5(3):499-511. Epub 2016/03/19. doi: 10.1002/mbo3.346.

56. Jurėnas D, Fraikin N, Goormaghtigh F, Van Melderen L. Biology and evolution of bacterial toxin-antitoxin systems. Nat Rev Microbiol. 2022;20(6):335-350. Epub 2022/01/04. doi: 10.1038/s41579-021-00661-1.

57. Kamruzzaman M, Wu AY, Iredell JR. Biological functions of type II toxin-antitoxin systems in bacteria. Microorganisms. 2021;9(6):1276. Epub 2021/07/03. doi: 10.3390/microorganisms9061276.

58. Sabrin A, Gioe BW, Gupta A, Grove A. An EmrB multidrug efflux pump in *Burkholderia thailandensis* with unexpected roles in antibiotic resistance. J Biol Chem. 2019;294(6):1891-1903. Epub 2018/12/14. doi: 10.1074/jbc.RA118.006638.

59. Zhang H, Ma Y, Liu P, Li X. Multidrug resistance operon *emrAB* contributes for chromate and ampicillin co-resistance in a *Staphylococcus* strain isolated from refinery polluted river bank. Springerplus. 2016;5(1):1648. Epub 2016/10/11. doi: 10.1186/s40064-016-3253-7.

60. Liang H, Mower JP, Chia CP. Functional prokaryotic-like deoxycytidine triphosphate deaminases and thymidylate synthase in eukaryotic social amoebae: Vertical, endosymbiotic, or horizontal gene transfer? Mol Biol Evol. 2023;40(12):msad268. doi: 10.1093/molbev/msad268.

61. Crawford MA, Henard CA, Tapscott T, Porwollik S, McClelland M, Vázquez-Torres A. DksA-dependent transcriptional regulation in *Salmonella* experiencing nitrosative stress. Front Microbiol. 2016;7:444. doi: 10.3389/fmicb.2016.00444.

62. Marković KG, Grujović MŽ, Koraćević MG, Nikodijević DD, Milutinović MG, Semedo-Lemsaddek T, et al. Colicins and microcins produced by *Enterobacteriaceae*: Characterization, mode of action, and putative applications. Int J Environ Res Public Health. 2022;19(18):11825. doi: 10.3390/ijerph191811825.

63. Vasicek EM, O'Neal L, Parsek MR, Fitch J, White P, Gunn JS. L-arabinose transport and metabolism in *Salmonella* influences biofilm formation. Front Cell Infect Microbiol. 2021;11:698146. Epub 2021/08/10. doi: 10.3389/fcimb.2021.698146.

64. Quistgaard EM, Löw C, Guettou F, Nordlund P. Understanding transport by the major facilitator superfamily (MFS): structures pave the way. Nat Rev Mol Cell Biol. 2016;17(2):123-132. doi: 10.1038/nrm.2015.25.

65. Drew D, North RA, Nagarathinam K, Tanabe M. Structures and general transport mechanisms by the major facilitator superfamily (MFS). Chem Rev. 2021;121(9):5289-5335. doi: 10.1021/acs.chemrev.0c00983.

66. Dik DA, Marous DR, Fisher JF, Mobashery S. Lytic transglycosylases: concinnity in concision of the bacterial cell wall. Crit Rev Biochem Mol Biol. 2017;52(5):503-542. doi: 10.1080/10409238.2017.1337705.

67. Kim H-S, Kim S-M, Lee H-J, Park S-J, Lee K-H. Expression of the *cpdA* gene, encoding a 3′,5′-cyclic AMP (cAMP) phosphodiesterase, is positively regulated by the cAMP-cAMP receptor protein complex. J Bacteriol. 2009;191(3):922-930. doi: 10.1128/jb.01350-08.

68. Bergman S, Andresen L, Kjellin J, Martinez Burgo Y, Geiser P, Baars S, et al. ProQ-dependent activation of *Salmonella* virulence genes mediated by post-transcriptional control of PhoP synthesis. mSphere. 2024;9(3):e00018-24. doi: 10.1128/msphere.00018-24.

69. Roux N, Spagnolo J, de Bentzmann S. Neglected but amazingly diverse type IVb pili. Res Microbiol. 2012;163(9-10):659-673. Epub 2012/10/30. doi: 10.1016/j.resmic.2012.10.015.

70. Little JI, Singh PK, Zhao J, Dunn S, Matz H, Donnenberg MS. Type IV pili of *Enterobacteriaceae* species. EcoSal Plus. 2024:eesp00032023. Epub 2024/01/31. doi: 10.1128/ecosalplus.esp-0003-2023.

71. Zhang XL, Tsui IS, Yip CM, Fung AW, Wong DK, Dai X, et al. *Salmonella enterica* serovar Typhi uses type IVB pili to enter human intestinal epithelial cells. Infect Immun. 2000;68(6):3067-3073. Epub 2000/05/19. doi: 10.1128/iai.68.6.3067-3073.2000.

72. Li C, Tyson GH, Hsu C-H, Harrison L, Strain E, Tran T-T, et al. Long-read sequencing reveals evolution and acquisition of antimicrobial resistance and virulence genes in *Salmonella enterica*. Front Microbiol. 2021;12:777817. doi: 10.3389/fmicb.2021.777817.

73. Zayas CL, Claas K, Escalante-Semerena JC. The CbiB protein of *Salmonella enterica* is an integral membrane protein involved in the last step of the de novo corrin ring biosynthetic pathway. J Bacteriol. 2007;189(21):7697-708. Epub 2007/09/11. doi: 10.1128/jb.01090-07.

74. Ross K, Varani Alessandro M, Snesrud E, Huang H, Alvarenga Danillo O, Zhang J, et al. TnCentral: A prokaryotic transposable element database and web portal for transposon analysis. mBio. 2021;12(5):e0206021. doi: 10.1128/mbio.02060-21.

75. Garrido V, Arrieta-Gisasola A, Migura-García L, Laorden L, Grilló MJ. Multidrug resistance in *Salmonella* isolates of swine origin: mobile genetic elements and plasmids associated with cephalosporin resistance with potential transmission to humans. Appl Environ Microbiol. 2024;90(5):e00264-24. doi: 10.1128/aem.00264-24.

76. Haniford David B, Ellis Michael J. Transposons Tn10 and Tn5. Microbiology Spectrum. 2015;3(1):MDNA3-0002-2014. doi: 10.1128/microbiolspec.mdna3-0002-2014.

77. Baharoglu Z, Bikard D, Mazel D. Conjugative DNA transfer iInduces the bacterial SOS response and promotes antibiotic resistance development through integron activation. PLoS Genet. 2010;6(10):e1001165. doi: 10.1371/journal.pgen.1001165.

78. Vasu K, Nagaraja V. Diverse functions of restriction-modification systems in addition to cellular defense. Microbiol Mol Biol Rev. 2013;77(1):53-72. Epub 2013/03/09. doi: 10.1128/mmbr.00044-12.

79. Foley S, L., Kaldhone P, R., Ricke S, C., Han J. Incompatibility group I1 (IncI1) plasmids: Their genetics, biology, and public health relevance. Microbiol Mol Biol Rev. 2021;85(2):e00031-20. doi: 10.1128/mmbr.00031-20.

80. Nekrasov SV, Agafonova OV, Belogurova NG, Delver EP, Belogurov AA. Plasmid-encoded antirestriction protein ArdA can discriminate between type I methyltransferase and complete restriction-modification system. J Mol Biol. 2007;365(2):284-297. Epub 2006/10/31. doi: 10.1016/j.jmb.2006.09.087.

81. Freire Martín I, AbuOun M, Reichel R, La Ragione RM, Woodward MJ. Sequence analysis of a CTX-M-1 IncI1 plasmid found in *Salmonella* 4,5,12:i:−, *Escherichia coli* and *Klebsiella pneumoniae* on a UK pig farm. J Antimicrob Chemother. 2014;69(8):2098-2101. doi: 10.1093/jac/dku098.

82. Gulig PA, Curtiss R, 3rd. Plasmid-associated virulence of *Salmonella typhimurium*. Infect Immun. 1987;55(12):2891-2901. Epub 1987/12/01. doi: 10.1128/iai.55.12.2891-2901.1987.

83. Oluwadare M, Lee MD, Grim CJ, Lipp EK, Cheng Y, Maurer JJ. The role of the *Salmonella spvB* IncF plasmid and its resident entry exclusion gene *traS* on plasmid exclusion. Front Microbiol. 2020;11:949. Epub 2020/06/06. doi: 10.3389/fmicb.2020.00949.

84. Silva C, Puente JL, Calva E. *Salmonella* virulence plasmid: pathogenesis and ecology. Pathog Dis. 2017;75(6). doi: 10.1093/femspd/ftx070.

85. Kidgell C, Pickard D, Wain J, James K, Diem Nga LT, Diep TS, et al. Characterisation and distribution of a cryptic *Salmonella* typhi plasmid pHCM2. Plasmid. 2002;47(3):159-171. Epub 2002/08/02. doi: 10.1016/s0147-619x(02)00013-6.

86. Octavia S, Sara J, Lan R. Characterization of a large novel phage-like plasmid in *Salmonella enterica* serovar Typhimurium. FEMS Microbiol Lett. 2015;362(8):fnv044. Epub 2015/03/22. doi: 10.1093/femsle/fnv044.

87. Greig DR, Bird MT, Chattaway MA, Langridge GC, Waters EV, Ribeca P, et al. Characterization of a P1-bacteriophage-like plasmid (phage-plasmid) harbouring $bla_{\text{CTX-M-15}}$ in *Salmonella enterica* serovar Typhi. Microb Genom. 2022;8(12):mgen000913. doi: 10.1099/mgen.0.000913.

88. Manyi-Loh C, Mamphweli S, Meyer E, Okoh A. Antibiotic use in agriculture and its consequential resistance in environmental sources: Potential public health implications. Molecules. 2018;23(4):795. doi: 10.3390/molecules23040795.

89. McMillan EA, Gupta SK, Williams LE, Jové T, Hiott LM, Woodley TA, et al. Antimicrobial resistance genes, cassettes, and plasmids present in *Salmonella enterica* associated with United States food animals. Front Microbiol. 2019;10:832. Epub 2019/05/07. doi: 10.3389/fmicb.2019.00832.

Supporting information

S1 Table. *Salmonella enterica* serovar Infantis strains screened for plasmids in this study.

Figure 1

Click here to access/download;Figure;FIG 1.tif

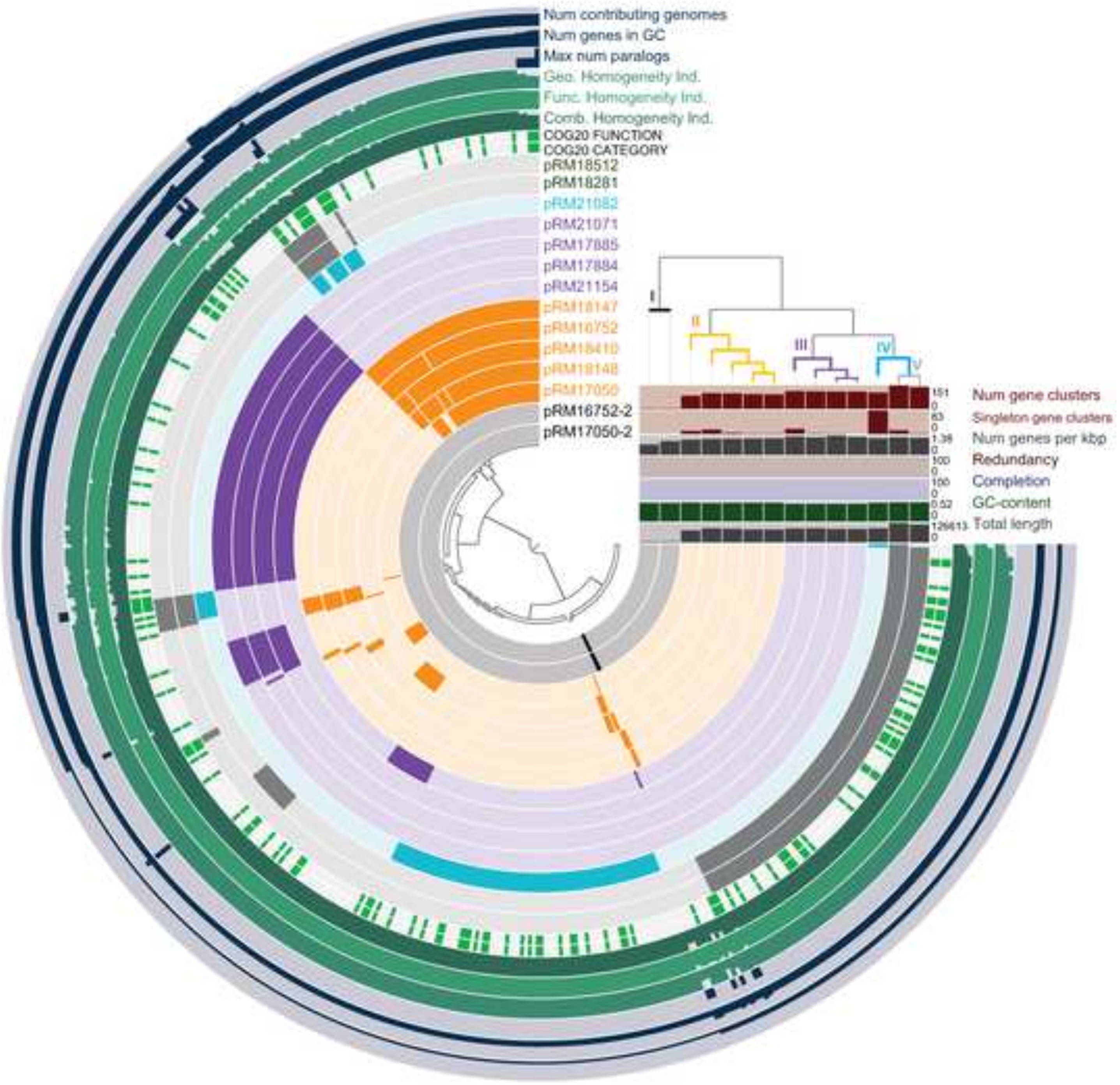

Figure 2

Click here to access/download;Figure;FIG 2.tif

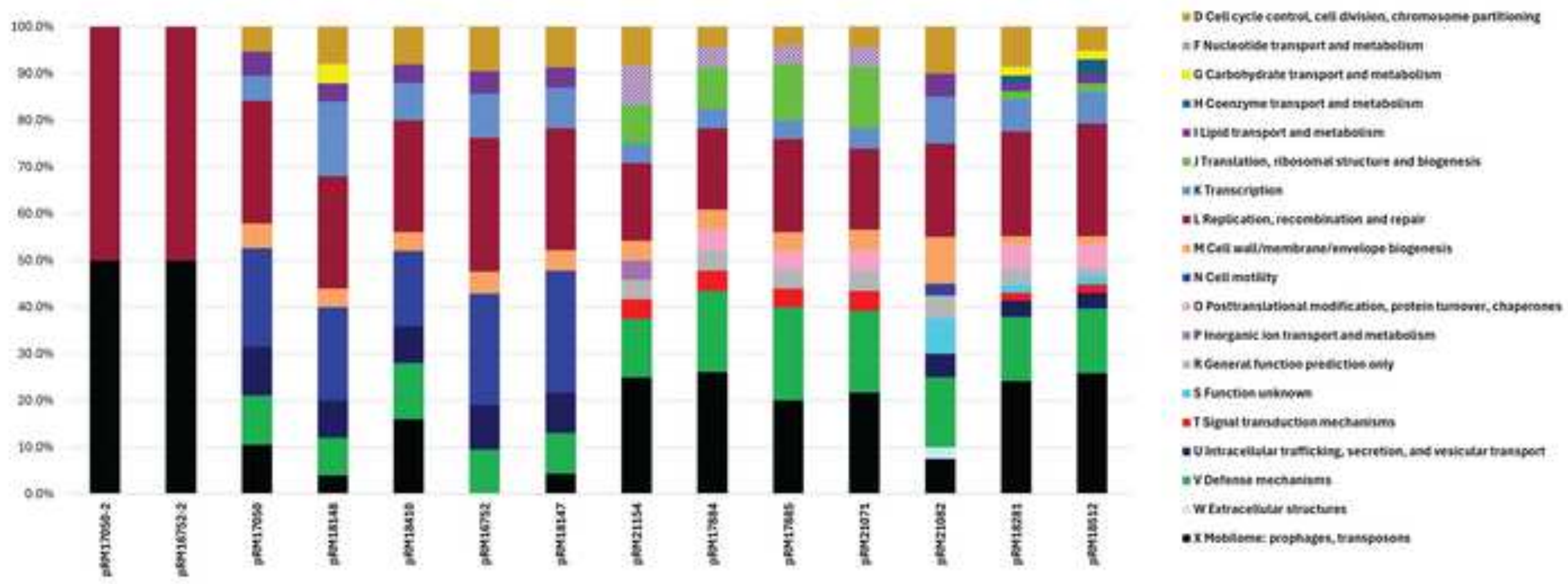

Figure 3

Click here to access/download;Figure;FIG 3.tif

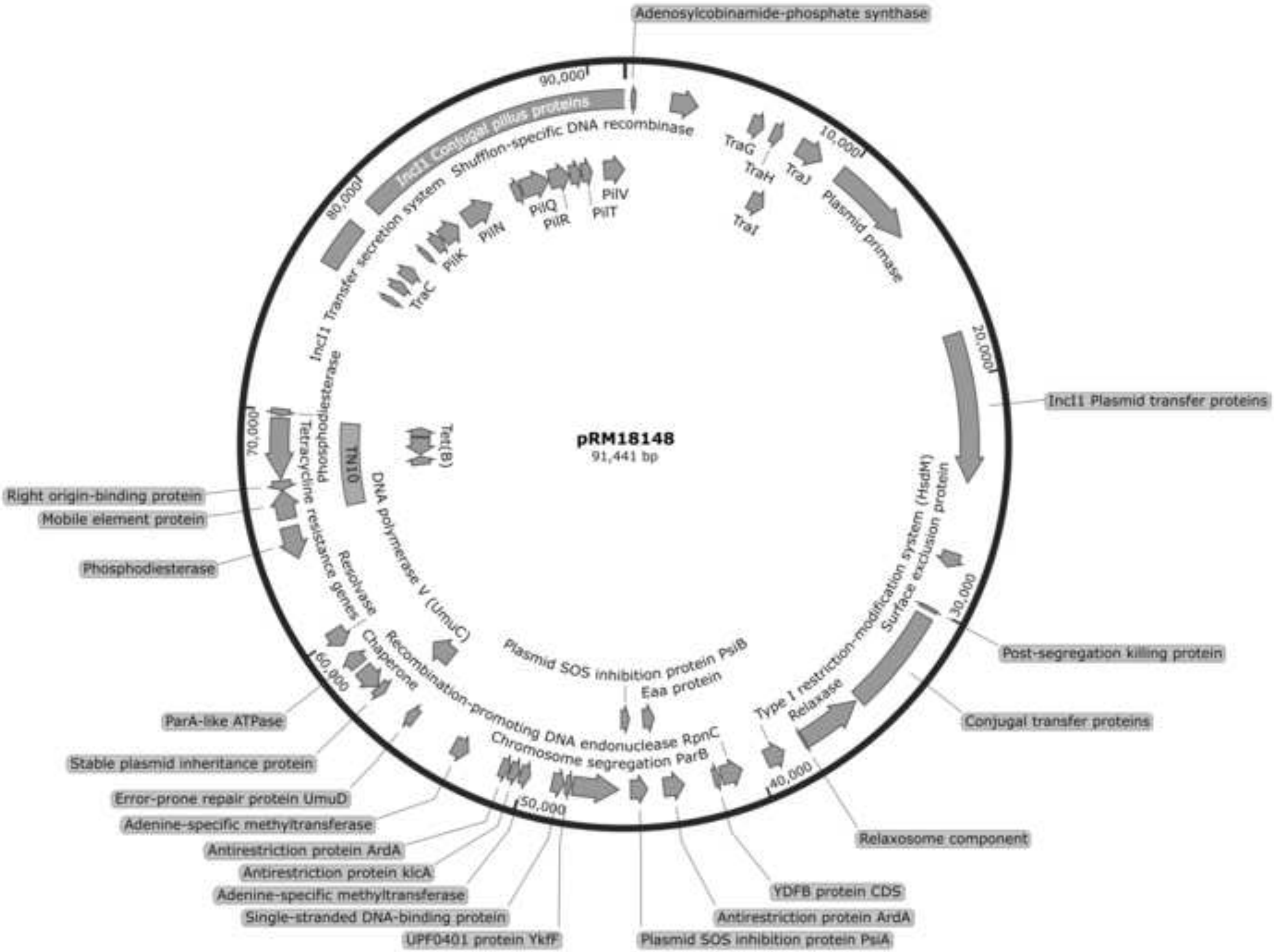

Supplementary Table

Click here to access/download
Supporting Information
S1_Table.docx
