# Supplementary material for: Plasmidome of Salmonella enterica serovar Infantis recovered from surface waters in a major agricultural region for leafy greens in California
Source: PLoS One. 2024 Dec 30;19(12):e0316466. doi: 10.1371/journal.pone.0316466 (PMC11684603; doi:10.1371/journal.pone.0316466)
Supplement: Attachment — Submitted filename: Responses to Comments.docx [file pone.0316466.s003.docx]

**RESPONSES TO EDITOR’S COMMENTS| MANUSCRIPT ID PONE-D-24-42911:**

**1. EDITOR’S COMMENT:** Please ensure that your manuscript meets PLOS ONE's style requirements, including those for file naming.

**AUTHORS’ REPLY:** The manuscript was formatted according to PLOS ONE’s guidelines.

**2. EDITOR’S COMMENT:** In your Methods section, please provide additional information regarding the permits you obtained for the work. Please ensure you have included the full name of the authority that approved the field site access and, if no permits were required, a brief statement explaining why.

**AUTHORS’ REPLY:**  In the Material and Methods section (lines 77-80), additional statements were inserted in the revised manuscript to indicate that the present study did not require permits issued for since all of the sampling sites were located on public lands within an approximate 20-mile radius of Salinas, California (USA). No specific permissions were required for sampling on the public lands, and these studies did not involve sampling any endangered or protected species.

**3. EDITOR’S COMMENT:** Please note that your Data Availability Statement is currently missing the direct link to access each database (GenBank accession numbers PQ365737-PQ365750).

**AUTHORS’ REPLY:** Given that the direct link to the sequences through the BioProject site is unavailable, the Data Availability Statement (lines 157-160) was edited in the revised manuscript by providing an alternate site (<https://www.ncbi.nlm.nih.gov/nuccore/>) to access the plasmid sequences in the NCBI nucleotide database.

**4. EDITOR’S COMMENT:** We note that you have included the phrase “data not shown” in your manuscript. Unfortunately, this does not meet our data sharing requirements. PLOS does not permit references to inaccessible data. We require that authors provide all relevant data within the paper, Supporting Information files, or in an acceptable, public repository. Please add a citation to support this phrase or upload the data that corresponds with these findings to a stable repository (such as Figshare or Dryad) and provide and URLs, DOIs, or accession numbers that may be used to access these data. Or, if the data are not a core part of the research being presented in your study, we ask that you remove the phrase that refers to these data.

**AUTHORS’ REPLY:** To address the Editor’s comment, the Material and Methods (lines 90-96) and Results (lines 325-329) sections are now included in the revised manuscript text on the analysis of the in vivo antimicrobial resistance against tetracycline, which was originally mentioned as “data not shown”.

**5. EDITOR’S COMMENT:** Please review your reference list to ensure that it is complete and correct.

**AUTHORS’ REPLY:** The reference list was reviewed to be complete and correct.

**6. EDITOR’S COMMENT** Restructure the manuscript. Separate the Results and Discussion sections. The Discussion section should not have any subtitles. In the Discussion, avoid repeating the results; instead, provide an interpretation of your findings, discuss their implications, and compare them with similar studies in the literature. Please do not cite any figures or tables in the Discussion section. The Conclusion section should provide a summary of the key findings from your study without any citations. Please ensure the Conclusion is focused on summarizing the main results and offering recommendations based on your findings. The recommendations should be provided in the final paragraph of the Conclusion section.

**AUTHORS’ REPLY:**  The manuscript was extensively revised, and the Results (pages 9-18) and Discussion (pages 18-23) are now in separate sections. The highlights of the present study and recommendations for future analyses have been included in the revised Conclusions section (page 24).

**7. EDITOR’S COMMENT:** Limitations: Highlight the key limitations of your study as the final paragraph of the Discussion section.

**AUTHORS’ REPLY:** The limitations of the current study were included after the Discussion section (page 24) by stating that the current study has provided fundamental information based on annotated sequence data. Given that only genomic data is provided in this study, additional comparative and functional analyses are thus needed to determine the expression patterns of the identified genes and dissect how their expression contributes to the virulence phenotypes.

**RESPONSES TO REVIEWER #1’S COMMENTS| MANUSCRIPT ID PONE-D-24-42911:**

**1. REVIEWER’S COMMENT:** Line 152: "Twelve strains were selected for sequence analysis." so, is it twelve strains out of the 34, 14 or the 20. please specify out of which group is the 12 selected from.
**AUTHORS’ REPLY:** An additional statement was inserted in the revised manuscript text (lines 166-167) to specify that the examined 12 Infantis strains were selected based on the high yield recovery of the detected large plasmids.

**2. REVIEWER’S COMMENT:** Lines 206-209: "As further demonstrated in the comparative analysis with the Anvi’o workflow (Fig 1), various homogeneity indexes were determined to measure the variation and frequency of their occurrence in the detected plasmid genes with an index of 1 being the highest value indicating no variation." please specify the indexes tool used for this study.
**AUTHORS’ REPLY:** To address the Reviewer #1’s question, the manuscript text was revised (lines 145-148) to indicate that the function in the Anvi’o workflow called “anvi-compute-gene-cluster-homogeneity” was employed to compute the geometric and functional homogeneity indexes, and these indexes provided measures of similarity in the detected plasmid genes with an index of 1 being the highest value to indicate no variation.

**3. REVIEWER’S COMMENT:** Did you have some limitation in this study? if yes please mention some.
**AUTHORS’ REPLY:** The limitations of the current study were included after the Discussion section (page 24) by stating that the current study has provided fundamental information based on annotated sequence data. Given that only genomic data is provided in this study, additional comparative and functional analyses are thus needed to determine the expression patterns of the identified genes and dissect how their expression contributes to the virulence phenotypes.

**4. REVIEWER’S COMMENT:** Did you have ethical approval for this study? please if yes is better if you include it in.
**AUTHORS’ REPLY:** In the Material and Methods section (lines 77-80), additional statements were inserted in the revised manuscript to indicate that the present study did not require permits issued for since all of the sampling sites were located on public lands within an approximate 20-mile radius of Salinas, California (USA). No specific permissions were required for sampling on the public lands, and these studies did not involve sampling any endangered or protected species

**5. REVIEWER’S COMMENT:**  What recommendation can you give based on the findings?
**AUTHORS’ REPLY:** Recommendations on the directions of future studies based on the results obtained in the present study are included in the Conclusions section (page 24). Recent report have documented that leafy greens have been previously a vehicle of foodborne illness, and surface waters can be a potential a route of transmission of bacterial pathogens. Therefore, subsequent phenotypical studies are aimed at further categorizing the virulence potential as well as the environmental fitness, adaptability and persistence of the examined S. Infantis strains, recovered from this important agricultural region for fresh produce.

**6. REVIEWER’S COMMENT:** Include the funding information for this study and possible conflict of interest if any.

**AUTHORS’ REPLY:** As indicated in the author guidelines for Plos One, the funding information and conflict of interest needed to be uploaded separately from the manuscript text by using the journal submission site.
