# Supplementary material for: Plasmidome of Salmonella enterica serovar Infantis recovered from surface waters in a major agricultural region for leafy greens in California
Source: PLoS One. 2024 Dec 30;19(12):e0316466. doi: 10.1371/journal.pone.0316466 (PMC11684603; doi:10.1371/journal.pone.0316466)
Supplement: Attachment — Submitted filename: Answers to 2nd Review Comments.docx [file pone.0316466.s004.docx]

1. **Restructure the Manuscript**
   - In the *Discussion* section, avoid repeating the results. Focus on interpreting your findings, discussing their implications, and comparing them with existing studies in the literature.
     - The Results and Discussion sections were edited. Results were removed from the Discussion, references were removed from Results.
2. **Results Section**
   - Remove all citations from the *Results* section. The *Results* should strictly state the findings, supported by accompanying tables and figures. Cite tables and figures as appropriate, but do not provide explanations or cite literature in this section.
     - Citations were removed from the Results section.
   - Specifically, citations found in lines 214 and 252–398 of the tracked manuscript version must be removed.
     - Citations were removed from the results section.
3. **Discussion Section**
   - Revise the *Discussion* section to include explanations, implications of findings, and comparisons with relevant literature. Do not restate the results in this section.
     - The Results and Discussion sections were edited. Results were removed from the Discussion, references were removed from Results.
4. **Other Issues**
   - Delete the phrase "see Materials and Methods" from line 245 in the tracked manuscript.
     - This phrase was deleted.
